# Preparation and Microstructural Characterization of Griseofulvin Microemulsions Using Different Experimental Methods: SAXS and DSC

**DOI:** 10.15171/apb.2017.034

**Published:** 2017-06-30

**Authors:** Eskandar Moghimipour, Anayatollah Salimi, Sahar Changizi

**Affiliations:** ^1^Nanotechnology Research Center, Ahvaz Jundishapur University of Medical Sciences, Ahvaz, Iran.; ^2^Department of Pharmaceutics, Faculty of Pharmacy, Ahvaz Jundishapur University of Medical Sciences, Ahvaz, Iran.

**Keywords:** Griseofulvin, Microemulsion, Microstructure, XRD, DSC, Stability

## Abstract

***Purpose:*** The objective of the present study is to formulate and evaluate a new microemulsion (ME) for topical delivery of griseofulvin.

***Methods:*** The solubilities of griseofulvin in different combinations of surfactant to co-surfactant (S/Co ratio) were determined. Accordingly, based on their phase diagrams, eight microemulsions were formulated and then evaluated with respect to their particle size, surface tension, viscosity, conductivity, zeta potential and stability. Their release behavior, Scanning Electron Microscopy (SEM), Differential Scanning Calorimetry (DSC), refractory index (RI), pH and Small-angle-X-ray scattering (SAXS) were also assessed.

***Results: ***The results indicated that the mean droplet size of the MEs ranged from 30.9 to 84.3 nm. Their zeta potential varied from -4.5 to -20.8. Other determined characteristics were viscosity: 254-381 cps, pH: 5.34-6.57, surface tension: 41.16- 42.83 dyne.cm^-1^, conductivity: 0.0442 – 0.111 ms.cm^-1^. The drug release was in the range of 22.4 to 43.69 percent. Also, hexagonal, cubic and lamellar liquid crystals were observed in SAXS experiments.

***Conclusion:*** It can be concluded that any alteration in MEs constituents directly affects their microstructure, shape, droplet size and their other physicochemical properties.

## Introduction

Griseofulvin is an anti-fungal agent used in the treatment of dermatophytic fungi among different species in the general Microsporum, Trychophyton and Epidermophyton.^1,2^ Also, it has been shown effective for treatment of several inflammatory skin diseases.^3^ Currently, however, because of numerous oral complications, its application is restricted. When used topically, the drug is directly transported to the location and forms a high concentration of drug in the lesion. Topically applied griseofulvin has been reported to be a better prophylactic agent than both miconazole and clotrimazole.^4,5^

In order to increase cutaneous drug delivery, ME vehicles have been more often employed over recent years.^6^ They consist of water, oil and a mixture of surfactants which makes them a homogeneous, optically isotropic and thermodynamically stable solution.^7-9^ ME formulations have been shown to be excellent for delivery of lipophilic and hydrophilic drugs via topical and systemic routes.^10^ They are suggested for oral, topical, dermal, transdermal, parentenal and pulmonary drug administration. The favorable drug delivery properties of MEs appear to be mainly attributed to its excellent solubility properties.^7,10,11^ Traditional emulsions and MEs are different essentially regarding their size of particles and also their stability.^12^ Due to their ability to enhance permeation of drugs, MEs have been designed to enhance transdermal absorption of drugs such as testosterone, dexamethasone, estradiol and celecoxib.^13-15^

One of the most important properties of MEs is that they improve therapeutic efficacy of the drug and permit reduction in the volume of the drug delivery vehicle, thereby minimizing toxic side effects. In some cases, the capacity of the ME to solubilize large amounts of lipophilics drugs can be advantageous as well.^16^The dispersed phase of a ME, aqueous or lipidic (o/w or w/o types) potentially serve as reservoir of both water-soluble and fat-soluble drugs which may be partitioned in external and internal phases.^13^ Various techniques have been employed to study the size, shape and interactions of ME droplets.^17^ Due to their differences in ME structures, they also show different patterns of release behavior of solubilized drug.^9^ MEspossess several advantages in drug delivery including modulation of release kinetics, absorption enhancement and decrease of drug toxicity.^18^ The objective of the present study was to formulate griseofulvin as a relatively stable ME for topical delivery.

Regarding the results of release studies, different structures and behaviors are suggested for microemulsions. Drug-loaded and drug-free MEs are characterized to evaluated drug delivery potential of different vehicles. The nature of drug loaded in MEs may affect their microstructure, phase behavior and stability. Despite their ease of preparation, identification of ME microstructures is relatively complex and needs a combination of several techniques. Although MEs are thermodynamically stable, their microstructure in the bicontinuous region is continuously changing, hence complicating structure determination. Utilizing many instrumental methods, such as determination of particle size, measurement of rheological properties, surface tension, electrical conductivity and also differential scanning calorimetry (DSC) may help to study MEs microstructures. Small angle x-ray scattering (SAXS) is also another instrumental technique for elucidating their structure. The nature of loaded drug affects many MEs properties including their phase behavior, stability and internal structures, so the effect of the drug on ME characteristics should be investigated.^19-23^

## Materials and Methods

Isopropyl myristate, oleic acid, span20, transcutol-P and tween80 were purchased from Merck Chemical company (Germany). Diethylene glycol monoethyl ether (Transcutol P), Caprylocaproyl macrogoglycerides (Labrasol), pleurol oleic were kindly gifted by GATTEFOSSE Company (France), and griseofulvin powder was obtained from Darou Pakhsh company (IR Iran). The effect of variables on different responses was assessed by experimental design using Minitab 17. Ternary phase diagrams were plotted Sigma plot 12.

### Griseofulvin Assay

A UV- spectrophotometry (BioWave II, WPA) at λmax of 294 nanometer was utilized to assay griseofulvin in samples.

### Screening of oils, surfactants and co-surfactants for microemulsions

The Solubility of griseofulvin in different oils (Isopropyl myristate, transcutol-P, oleic acid), surfactants (Labrasol, Tween 80, Span 20) and cosurfactants (Pleurol Oleic, Propylene glycol) was determined by dissolving an extra amount of griseofulvin in 5 mL of each oil, surfactant and cosurfactant. The samples were mechanically agitated by means of a shaking water bath functioning at 200 strokes per min (spm) for 72 h at 37± 0.5 °C to reach equilibrium. After equilibration,the samples were centrifuged at 10000 rpm for 30 min to exclude the undissolved drug. In the next step, the clear supernatants were filtered through a polytetrafluoroethylene membrane filter (φ= 0.45 µm) and the filtrates were assayed using UV spectrophotometry. Their solubilites were measured in triplicate.^24^

### Phase Study

Pseudo-ternary Phase diagrams of unloaded MEs were prepared to investigate the concentration range of the components for the existing boundary of MEs and three phase diagrams were organized with the 3:1, 4:1, and 5:1 weight ratios of (Labrasol/Span 20) Pleurol oleic respectively. For each phase diagram, the surfactant mixture was supplemented into the oil phase (Oleic acid-Transcutol P) (10:1) then mixed at the weight ratios of 9:1, 8:2, 7:3, 6:4, 5:5, 4:6, 3:7, 2:8, and 1:9. Using a magnetic stirrer, the samples were mixed, robustly and diluted dropwise with double distilled water at 25±1°C. The samples were classified as microemulsions with transparency character.^25^ The SigmaPlot®12.0 was utilized to determine their microemulsion region.

### Formulation of Griseofulvin MEs

Several parameters affected the final properties of microemulsions. After the ME region in the phase diagram was obtained, Full factorial design was used concerning the 3 variables at 2 levels for preparing eight formulations. Major variables play a role in determining ME’s characters including surfactant/co-surfactant ratio (S/C), the percent of of water (%W), and percent of oil (%Oil). Eight formulations having maximum and minimum levels of oil (30% and 5%), water (3%, 5%), and S/Co mixing ratio (3:1, 5:1) were selected (Table 1). Different MEs were selected in the pseudo-ternary phase diagram with 3:1, and 5:1 weight ratio of span20- Labrasol/Pleurol Oleic. Griseofulvin (0.2%) was added to oil phase and then S/Co mixture and a suitable amount of double distilled water were added to the mixture drop wise and continued by stirring the mixtures at ambient temperature until a uniform mixture was obtained.^26^

### Differential Scanning Calorimetry 

Differential scanning calorimetry (DSC) measurements were calculated using a Metller Toldo DSC1 star ® system fitted with refrigerated cooling system. Approximately 5-15 mg of each ME samples were weighted into hermetic aluminum pans and swiftly wrapped to stop water evaporation from ME samples. Concurrently, an empty hermetically closed pan was employed as a reference. ME samples were exposed in a temperature varying from +30°C to - 50°C (scan rate: 10°C/min). To guarantee precision and reliability of data, DSC instrument was calibrated and assessed under the conditions of use by indium standard. Transitions of enthalpy quantities (∆H) were computed from endothermic and exothermic peaks of thermograms.^27^

### Scanning Electron Microscopy (SEM)

Scanning electron microscopy was utilized to characterize internal microstructure of micro emulsions. SEM of MEs were analyzed by LEO 1455VP, Germany.^15^

### Measurement of Zeta Potential

Zeta potential of MEs were determined by Zetasizer (Malvern instrument Ltd ZEN3600, UK). Microemulsion formulations were placed in clean disposable zeta cells, and results were documented. Before placing the fresh sample, cuvettes were washed by methanol and rinsed using the sample to be measured prior to each experimentation.^19^

### Particle Size Measurements

The droplet size of MEs was measured at 25±1 °C by SCATTER SCOPE 1 QUIDIX (South Korea). Then was no sample dilution before the experiment.^26^

### Viscosity Determination

The ME samples viscosity was measured at 25±1°C using a Brookfield viscometer (DV-II + Pro Brookfield, USA) via spindle no. 34. with shear rate of 100 rpm. A 10 mL volume sample was used for viscosity measurements.^27^

### Electrical Conductivity Measurements

Electrical conductivity of MEs were measured using a conductivity tester (Metrohm Model 712). This was achieved by means of conductivity cell (with a cell constant of 1.0) containing two platinum plates detached by preferred distance and having liquid between the platinum plates performing as a conductor.^28,29^

### pH Measurement 

The ostensible pH values of the ME samples were specified at 25±1 °C by pH meter (Mettler Toledo, Switzerland). All of the experiments were performed three times.^30^

### Surface Tension Determination

The surface tensions of MEs were measured at 30±1°C by DU Nouy ring torsion balance (White Electrical Instrument Company (Model 83944E) fitted with platinium ring. A 5 mL volume sample was used for surface tension measurements.

### Stability of Drug–Loaded MEs

MEs were analyzed for their stability by temperature and centrifuge stability tests. They were stored in different temperature conditions (4°C, 25°C, 37°C and75% ± 5% RH for six months) according to ICH guidelines and then inspected by monitoring time- and temperature-dependent physicochemical alterations, including phase separation, flocculation, precipitation and particle size changes. Also after centrifugation at 15000 rpm for 30 minutes at 25±1°C in a high speed brushless apparatus ((MPV-350R, POLAND), the samples were visually inspected to detect any phase separation.^26,27^

### Release Study

Especially designed Franz diffusion cells having contacted area of 3.46 cm2 were used to evaluate the drug release from different formulations. Prior to each experiment, the cellulose membrane was placed in double distilled water at 25°C for 24 hrs to achieve complete hydration. Then, it was mounted between donor and receptor compartments. Griseofulvin samples (5g ME) were accurately weighed and placed on the membrane. 25 ml phosphate buffer solution (PBS) pH 7 and methanol (2:1 ratio) was utilized as receptor medium. The solutions were continuously stirred during the experiments. At definite time intervals (0.5, 1,2,3,4,5,6,7,8 and 24 h), 2 ml sample was removed from receptor compartments and then analyzed spectrophotometrically (Bio Wave II, WPA) at 294 nm for the drug content. To maintain sink conditions, an equal volume of the fresh receptor solution was added to the receptor chamber. The cumulative percentage of released drug was plotted versus time and their behavior was described by fitting on different kinetic models. The maximum r2 was considered as the most probable mechanism.^18,24^

### Small – Angle X-ray Diffraction (SAXS) 

Philips PC-APD diffractometer (Xpert MPD) equipped with Goniometer type pW3050/e-2e and Ni-filtered Co Kα radiation (d = 1.78897˚A) at operating power generator 40KeV and 30 mA, ranged from 1.11 to 9.9˚2θ and rate of scanning of 0.02˚/sec was used to depict griseofulvin MEs SAXS. The MEs were put in a spinner phase in a thermally measured sample holder centered in the X-radiation beam. Miniprop detector was utilized to gather intensity data. X-ray scattering was done at 25±1°C and each formulation was scanned three times.^21,28^

### Statistical Methods

All the tests were performed in triplicate, and data were expressed as the average value ± SD. The data were statistically analyzed by one-way analysis of variance (ANOVA), and P < 0.05 was considered as significant with 95% confidence intervals.

## Results and Discussion

### Griseofulvin Solubility 

The drug performance in a ME system is generally affected by its solubility. Griseofulvin solubility in the various Oils, Surfactants and Cosurfactants was detected by the shake-flask method.^24^ Values of equilibrium solubility are tabulated in Table 1.

**Table 1:** The equilibrium Solubility of Griseofulvin in Various Oils, Surfactants and Cosurfactants (Mean ± SD, n = 3)

**Phase type**	**Excipient**	**Solubility (mg/ml)**
oil	Oleic acid	2.66 ± 0.15
	Oleic acid + transcutol P	3.61 ± 0.04
	Isopropyl Myristat	2.01 ± 0.07
	IsopropyMyristat + transcutol	3.50 ± 0.20
surfactant	Tween 80	3.06 ± 0.30
	Span 20	3.09 ± 0.10
	Labrasol	4.36 ± 0.15
co-surfactant	Pleurol Oleic	2.60 ± 0.10
	Propylene glycol	1.00 ± 0.01

The solubility of griseofulvin was maximum in Oleic acid:Transcutol P (10:1) (3.61 ± 0.04mg/ml) as compared to other oils. Furthermore, the maximum solubility of griseofulvin in surfactants was found in Labrasol (4.36 ± 0.15mg/mL), and Span 20 (3.09 ± 0.10mg/mL) and cosurfactant, Pleurol Oleic (2.60 ± 0.10 mg/mL).

### Pseudo-Ternary Phase Diagram

**Figure 1:**
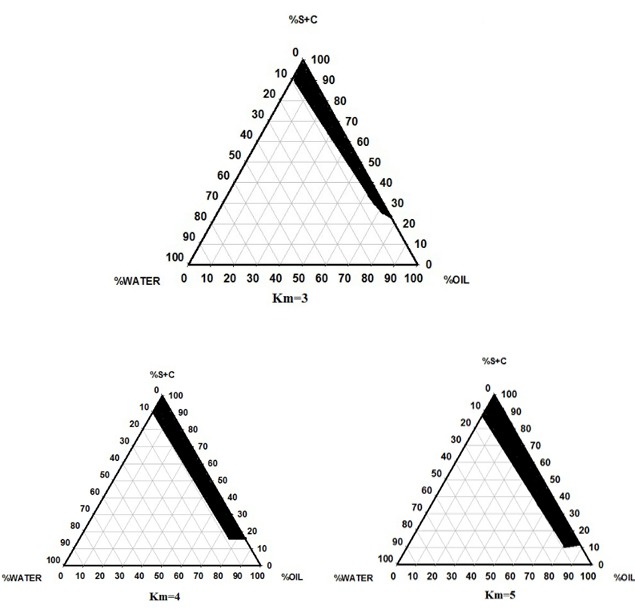
The Pseudoternary Phase Diagrams of the Oil-surfactant/Cosurfactant Mixture-water System at the 3:1, 4:1, and 5:1 Weight Ratio or Labrasol/Tween 20/ Pleurol Oleic at Ambient Temperature, Dark Area Show Microemulsions Zone.

Pseudo-ternary phase diagrams, presented in Figure 1, were plotted to discover the presence of different ME regions. It appears that phase behavior is contingent on co-surfactant and surfactant quallities. The weight ratio of surfactant/cosurfactant mixture (Km) is a significant factor influencing phase behavior of ME. An increase in ME area was observed with increasing of relative concentration of surfactant.^26^ The phase diagrams showed that ME region extended with large quantity in the weight ratio of surfactant/cosurfactant (km = 3-5). Based on visual inspection, the remaining section of the phase diagram signifies conventional and turbid emulsions.

### Characterization of the Griseofulvin–Loaded Microemulsion Preparations

Eight different MEs were chosen from the pseudo-ternary phase diagram with 3:1, and 5:1 weight ratio of Labrasol -Span 20/Pleurol Oleic. The composition of selected MEs is shown in Table 2. The zeta potential, viscosity, mean particle size, polydispersity index (PI), pH, surface tension, conductivity, and refractive index of Griseofulvin microemulsions are presented in Table 3. The ME samples in this study revealed the average viscosity range (270.2 ± 1.23 cps–386.4±1.43cps), zeta potential (-5.97to -20.82mv), pH value (5.34 to 6.57), and particle size (22.4 -84.3 nm). ANOVA revealed that viscosity, pH and average particle size are significantly correlated with oil percentage. It seems that the mean particle size, viscosity and pH are increased with less percentage of oil phase in some of MEs. Also, ANOVA indicated that correlation between zeta potential and independent variables (%Water) is significant (P < 0.05). In some microemulsions, the zeta potential increased with decrease of water phase.

**Table 2:** Composition of Selected Griseofulvin - Loaded Microemulsions

**Formulation**	**Factorial**	**S/C**	**% Oil**	**%(S/C)**	**% Water**
MEG-1	+++	5:1	30	65	5
MEG-2	‏- + +	5:1	30	67	3
MEG-3	‏+ - +	5:1	5	90	5
MEG-4	‏- - +	5:1	5	92	3
MEG-5	‏- - -	3:1	5	92	3
MEG-6	‏+ - -	3:1	5	90	5
MEG-7	‏- + -	3:1	30	65	3
MEG-8	‏+ + -	3:1	30	65	5

The refractive index (RI) of the formulations was determined at 1.46 which is close to oil phase signifying that formulations exhibit water-in-oil structures. ANOVA revealed that there was no significant correlation between water content and RI. Due to conductivity potential of aqueous phase, oil in water microemulsion exhibit higher conductivity values than the W/O microemulsions.^29^ It was shown that the conductivity of Griseofulvin samples was in the range of 0.0564-0.102ms/cm.

**Figure 2:**
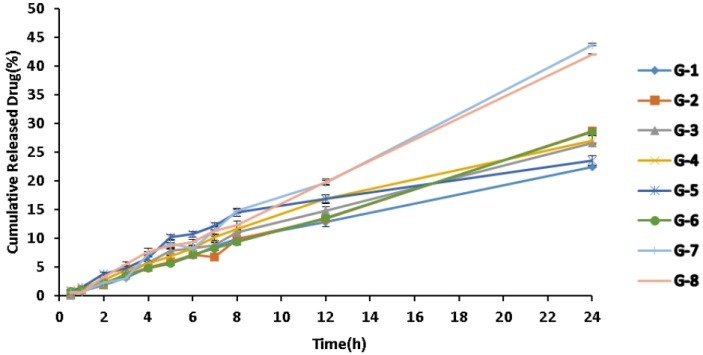
In vitro Release Profile of MEs Formulation of Griseofulvin

It was shown that all of the MEs have proper characteristics regarding their homogeneity and six-months duration stability. Average droplet sizes at the beginning and after six months of storage of the MEs showed no significant difference (p>0.05). Visually inspection during the storage showed no precipitation, phase separation or flocculation. Centrifugation of the samples at 15000 rpm for 30 minutes caused no phase separation and the MEs remained homogenous during and after examination. As shown in Figure 2, 43.697 % of griseofulvin loaded in MEG-7 is released during the first 24 hours of experiment and it exhibited zero-order kinetics. Percentage of the released drug and the release kinetics of MEs are shown in Table 4.

### Differential Scanning Calorimetry (DSC)

**Figure 3:**
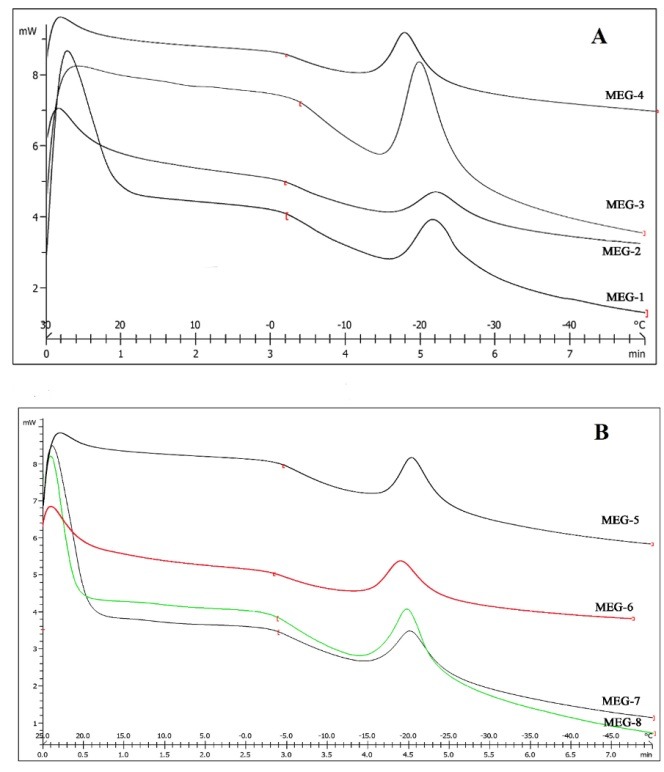
DSC cooling thermograms of griseofulvin MEs. (A, B)

Figure 3 shows DSC cooling thermograms of griseofulvin MEs. Table 5 shows enthalpies and cooling transition temperatures of MEs. Cooling graphs indicated the presence of bound water and bulk water (free water) in -18 to -21.5°C and 0 to -3°C, correspondingly.

The obtained results of DSC experiment gives useful information about water state and chemical and physical alterations that affects exothermic or endothermic processes in heat capacity.^23,30^ DSC studies were employed for aqueous mixed behavior of MEs and differentiation between bound (interfacial) water and bulk (free).^23^ Differential Scanning Calorimetry (DSC) has been utilized to calculate heat flow that is associated with transitions in materials as a function of temperature. In cooling graphs of the MEG-1, DSC thermograms demonstrated two exothermic peak at around-2°C -21.5°C which shows that the freezing of free and bulk water in this formulation and inMEG-2 implies two exothermic peaks at around -1°C (bulk water) and -20°C (bound water). In cooling graphs of MEG-3 and MEG-4 observed two exothermic peaks at-1°C, -3°C (free water)and -20°C,-18°C (bound water), respectively.

**Table 3:** pH, Viscosity, Conductivity, Zeta Potential, Refractive Index,particle size, PI and surface tension Selected Griseofolvin Microemulsions (Mean ± SD, n = 3)

**Formulation**	**pH**	**Viscosity, cps**	**Conductivity,** **ms/cm**	**Zeta Potential,** **mV**	**Refractive** **Index**	**Particle size(nm)**	**Poly dispersity index**	**Surface tension** **(dyne/cm)**
MEG-1	5.34±0.06	270.2±1.23	0.0564±0.001	-18.6±0.5	1.4604±0.12	36.8±.08	0.387±0.028	42.5±1.23
MEG-2	5.70±0.12	254.5±1.54	0.102±0.003	-9.14±0.3	0.15‏ ± 1.4603	22.4 ± .08	0.381±0.011	42.5±1.38
MEG-3	6.45±0.08	350.3±0.95	0.091±0.002	-5.97±0.2	1.4613±0.22	57.3 ± .09	0.362±0.017	41.6±0.98
MEG-4	6.45±0.18	363.5±1.34	0.0564±0.001	-4.54±0.6	1.463±0.22	60.7 ± .07	0.384±0.011	4303±1.35
MEG-5	6.40±0.08	386.4±1.43	0.111±0.002	+6.71±0.1	1.4649±0.18	84.3 ± 0.8	0.373±0.017	42.3±1.17
MEG-6	6.57±0.13	368.0±0.98	0.0442±0.001	-12.7±0.7	1.4629±0.24	79.8 ± .07	0.382±0.011	42.5±1.21
MEG-7	5.61±0.13	281.8±1.32	0.0777±0.001	-14.2±0.8	1.4619±0.17	67.7 ±0.06	0.380±0.012	41.3±1.27
MEG-8	5.36±0.09	280.1±1.62	0.0964±0.001	-20.82±1	1.4609±0.19	30.9 ± .12	0.388±0.014	42±1.24

**Table 4:** Percent Release and Kinetic Model Release of Selected Microemulsions (Mean ±SD, n=3)

**Formulation**	**Kinetic model Release**	**R** ^ 2 ^	**Release,%**
MEG-1	log wagner	0.9984	22.401±1.05
MEG-2	Zero	0.9956	28.637±1.22
MEG-3	First	0.9927	26.61±1.029
MEG-4	First	0.9915	27.066±2.36
MEG-5	log wagner	0.9857	23.564±1.83
MEG-6	Zero	0.9992	28.546±1.25
MEG-7	Zero	0.9927	43.697±3.14
MEG-8	Zero	0.9959	41.943±1.3

DSC graphs of MEG-5 and MEG-6 showed bulk water (0°C) and bulk water (-18°C and -19°C) respectively. DSC thermograms of MEG-7 and MEG-8 demonstrated two peaks at -3°C (bulk water) and -20°C (bound water). According to ANOVA results, specifically for MEG-1, MEG-2, MEG-7 and MEG-8, a significant correlation (P˂0.05) was found between the bound water melting transition temperature and independent variables, so that any increase in oil amount significantly decreased the temperature. Also, the independent variables in MEG-1, MEG-2, MEG-7 and MEG-8 formulations significantly affected enthalpy of exothermic peak of free water (P˂ 0.05); e.g., the enthalpy was increased due to increase of oil percentage.

### Scanning Electron Microscopy

**Figure 4:**
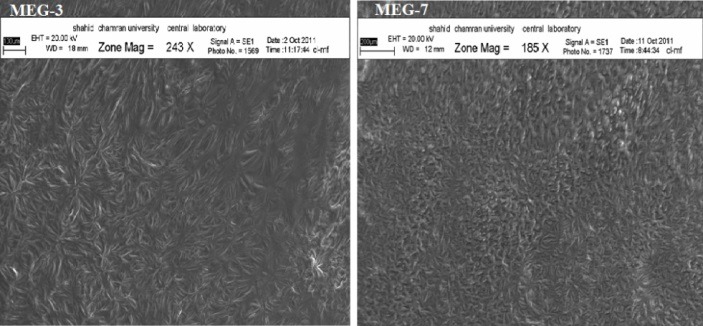
SEM images of MEG-3 and MEG-7

SEM of the MEs are shown in Figure 4. According to the results, the droplets in O/W and W/O phases were spherical and/or as irregular ellipsoid shapes, while there was no regular shape in bicontinuous ME phase in which labyrinthic networks are formed by intertwining of water and oil domains.^31^ Figure 3 shows the SEM images ofMEG-3 and MEG-7.

**Table 5:** Transition temperature and enthalpy of Griseofulvin MEs

**Formulation**	**Tm (°C)**	**∆H (m**_J_ **/mg)**
MEG-1	-21.5±0.4	3.8265±0.15
	-2±0.1	0.9668±0.01
MEG-2	-22±0.3	2.5628±0.11
	-1±0.05	0.4497±0.05
MEG-3	-20±0.3	2.8428±0.13
	-3±0.15	0.4354±0.04
MEG-4	-18±0.2	2.7070±0.12
	-1±0.03	0.3739±0.04
MEG-5	-18±0.1	2.1339±0.1
	0	0.0495±0.01
MEG-6	-19±0.15	2.7835±0.14
	0	0.3049±0.06
MEG-7	-20±0.3	0.9359±0.01
	-3±0.06	3.8209±0.15
MEG-8	-20±0.8	0.8754±0.02
	-3±0.03	3.5442±0.13

### Small-angle X-ray Scattering(SAXS)

In the current study, Small-angle X-ray Scattering was employed to survey the microstructure of MEs. SAXS results of the ME samples are presented in Figure 4 and Table 6.

Small-angle X-ray Scattering (SAXS) techniques are utilized by more than a few researchers to gain insights about droplet size and microstructure of MEs.^21,32,33^ With X-ray scattering experiments, typical interferences are produced from an ordered microstructure. A representative pattern of interference develops because of specific repeat distances of the correlated interlayer spacing d. by Bragg’s equation. The periodic interlayer spacing (d) was calculated by the Bragg’s equation n λ = 2dsinθ, where λ is the wave length of the X-ray, n is an integer and sates the order of the interference and, θ is the angle under which interference occurs.^34^ The interlayer spacing of crystalline liquids may be exactly determined by SAXS method, which calculates either interferences between the spacings or the sequences of interferences.^33,35^ The sequence of the interferences for Lamellar, Hexagonal, Cubic I and II, liquid crystals gives 1: ½: 1/3:1/4….., 1: 1/√3: 1√4: 1√7 …., 1: 1/√2: 1/√3: 1/√4 ….. and 1: 1/√4: 1/√5: 1/√6 ….. technique of X-ray diffraction, respectively.^36^

**Table 6:** d- Spacing amount (^0^Angstrum), d-spacing ratios and detected microstructures in Griseofulvin ME formulations

**Formulation**	**d-spacing amount(** ^ 0 ^ **A)**	**d-spacing ratios**	**microstructure**
MEG-1	37.007,26.35,21.14,17.71	1:1/√2:1/√3:1/√4	Cubic
MEG-2	35.817,26.626,20.303,14.345	1:1/√2:1/√3:1/√6	Cubic
MEG-3	33.83,19.60,16.05,13.43	1:1/√3:1/√4:1/√7	Hexagonal
MEG-4	36.22,25.69,19.90,18.08	1:1/√2:1/√3:1/√4	Cubic
MEG-5	40.84,22.834,19.237,15.949	1:1/√3:1/√4:1/√7	Hexagonal
MEG-6	46.80,22.733,16.20,12.93	1:1/2:1/3:1/4	Lamellar
MEG-7	50,37,29.206,25.43	1:1/√2:1/√3:1/√4	Cubic
MEG-8	37.826,26.357,20.384,18.085	1:1/√2:1/√3:1/√4	Cubic

**Figure 5:**
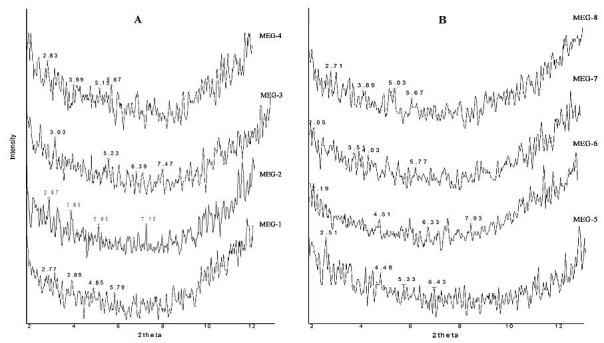
Small angle X-ray scattering curves for griseofulvin microemulsions.(A, B)

Figure 5 and Table 6 show the impact of independent variables and the drug on diffraction features and microstructure of the formulations. Various internal structures including, cubic, lamellar and hexagonal are detected in different MEs. Also, for MEG-3 and MEG-6 which were contained equal volumes of oil and water, bicontinuous phases were identified (Figure 4). For MEG-3 and MEG-7, hexagonal and cubic microstructures were detected, respectively.

The maximum water content and the least oil percentage in MEG-6 led to a lamellar microstructure, the finding that is not consistent with Raman et al. reports.^19^ By lowering surfactant and co-surfactant percentage in MEG-7 and MEG-8, the lamellar structure (MEG-6) changed to cubic form. MEG-3 and MEG-5 formulations that consisted of the least amount of oil content exhibited hexagonal microstructures, suggest that lowering the oil amount may lead to highly ordered structures. The finding is consistent with the previous reports.^37^ Long-range positional order in two or three dimensions is associated with hexagonal and cubic structures, respectively.^38^ While the drug distribution in hexagonal phase is analogous to that in cubic phase, it consists of cylindrical micelles that are arranged in a hexagonal order, and unlike the cubic arrangement, the water channels are completely closed. The cubic order, which is the principal microstructure in griseofulvin–loaded MEs is composed of two continuous but non-intersecting water channels that are separated by a lipid bilayer.^39^ Such a structure may be useful for entrapment of hydrophobic, hydrophilic and also amphiphilic molecules. Hydrophilic drugs are positioned near the lipid polar head group or in water channels, while griseofulvin as a lipophilic molecule locates in lipid bilayer. The main position for amphiphilic drugs is phase interfaces.^40^

## Conclusion

Internal structure of microemulsions such as cubic, lamellar and hexagonal liquid crystal structures were evaluated by different methods including SAXS. Any alteration in water, oil and surfactant content of microemulsions significantly changed their structures. The results indicated that the presence of liquid crystal structure may affect their release, viscosity and other characteristics of the formulations. DSC technique revealed the presence of bound and free water in microemulsions.

## Acknowledgments

This paper is extracted from Pharm. D. thesis (Changizi, S), and financial support was provided by Ahvaz Jundishapur University of Medical Sciences. The authors are very thankful to Faratin company manager (Taheri, M, Iran) for providing gratis samples of Labrasol, Transcutol P, Pleurol Oleic from GATTEFOSSE (France).

## Ethical Issues

Not applicable.

## Conflict of Interest

The authors declare no conflict of interest.
